# Case series: Potential use of guanfacine for safer management of behavioral disturbances in patients with dementia

**DOI:** 10.1002/pcn5.70122

**Published:** 2025-05-19

**Authors:** Jeong Hoo Lee, Joji Suzuki

**Affiliations:** ^1^ Brigham and Women's Faulkner Hospital Boston Massachusetts USA; ^2^ Harvard Medical School Boston Massachusetts USA; ^3^ Brigham and Women's Hospital Boston Massachusetts USA

**Keywords:** agitation, behavioral disturbance, dementia, guanfacine, neuropsychiatry

## Abstract

**Background:**

Behavioral and psychological symptoms of dementia (BPSD), including irritability, agitation, and anxiety, are common and cause significant distress for both patients and caregivers. These symptoms are linked to caregiver distress and early institutionalization, with disruptive behaviors like aggression often leading to emergency care. While antipsychotics have been used to manage BPSD, they are associated with serious side‐effects. There is a critical need for alternative effective and safer treatments that can reduce reliance on antipsychotics. In 2022, the therapeutic use of guanfacine to manage these symptoms in elderly dementia patients was suggested in two cases.

**Case Presentation:**

We present two additional clinical cases in which guanfacine was potentially effective in addressing behavioral disturbances in dementia patients at low doses and over a short period of time.

**Conclusion:**

Guanfacine may serve as a beneficial and well‐tolerated option for managing behavioral issues in dementia patients, potentially reducing the reliance on antipsychotics and minimizing the risk of related side‐effects.

## BACKGROUND

Behavioral and psychological symptoms of dementia (BPSDs) are common across all forms of dementia. The most prevalent symptoms include irritability, agitation, and anxiety, which contribute to significant distress and poor quality of life for both patients and caregivers.^1^, ^2^, ^3^ BPSDs often correlate with caregiver distress and are a leading cause of early institutionalization.^4^, ^5^, ^6^ Disruptive behaviors, such as aggression and screaming, are particularly burdensome and frequent reasons for emergency care.^1^, ^7^, ^8^, ^9^, ^10^ Prior to 2023, no Food and Drug Administration (FDA)‐approved medications were available for managing neuropsychiatric symptoms in Alzheimer's disease. However, brexpiprazole is now approved for treating agitation associated with dementia.^11^, ^12^ While antipsychotics and mood stabilizers have been commonly used for BPSD, they carry significant side‐effects, and atypical antipsychotics have a black‐box warning for increased mortality and cerebrovascular events in elderly patients with dementia.^13^

In 2022, the successful use of guanfacine to treat irritability, impulsivity, and agitation in elderly dementia patients was suggested in two cases.^14^ Here, we present two additional cases where guanfacine was potentially effective for similar symptoms, minimizing the use of antipsychotic medications and offering safer management of the patients.

## CASE PRESENTATION

### Case 1

An 86‐year‐old female with Alzheimer's disease, hypertension, type 2 diabetes, cataracts, osteoarthritis, heart failure (ejection fraction of 57%), atrial fibrillation (on apixaban), and chronic kidney disease presented to the hospital from a nursing home for behavioral agitation and was admitted to the medicine service. Her dementia diagnosis was first suspected about 8 years previously, and she had been started on donepezil. Over time, her condition progressed, and 3 years later, she could no longer perform activities of daily living (ADLs). At that time, she scored 22/30 on the Montreal Cognitive Assessment (MoCA), and a brain MRI showed findings consistent with Alzheimer's disease. A month prior to this hospitalization, the patient had fallen and suffered a mild humeral fracture, becoming delirious during hospitalization. Her behavioral outbursts had increased since that episode.

The day before her presentation, she became agitated and non‐redirectable for several hours. A computed tomography (CT) head scan showed no acute intracranial abnormalities, and her brain MRI was again consistent with Alzheimer's disease (AD). Other medical workups, including complete blood count (CBC), basic metabolic panel (BMP), urinalysis (UA), and chest X‐ray, were unremarkable. Upon admission, the patient frequently screamed and was noncommunicative when asked about her behavior. She remained confused and disoriented throughout the admission without prominent waxing and waning of mental status. On hospital day (HD) 1, the patient received olanzapine 5 mg iv (prn for agitation), olanzapine 5 mg im (prn for agitation), and quetiapine 25 mg po (prn for anxiety/screaming). On HD 2, she received quetiapine 62.5 mg po (prn 50 mg and 12.5 mg doses for anxiety/screaming). On HD 3, she received haloperidol 5 mg iv (prn for agitation), olanzapine 5 mg iv (prn for agitation), and quetiapine up to 100 mg po (prn 50 mg, 25 mg, 12.5 mg doses for anxiety/screaming/agitation). These medications had mild to moderate effects in calming her acutely but often sedated her. She also received scheduled oxycodone 5 mg po daily to address any potential pain component contributing to her screaming, though this had minimal effect. Other medications included apixaban 5 mg bid, fludrocortisone 0.1 mg qd, furosemide 20 mg qd, gabapentin 100 mg bid, spironolactone 25 mg qd, memantine 5 mg bid, midodrine 10 mg tid, and metoprolol 25 mg tid, all of which the patient had already been taking prior to the admission.

The psychiatry team was consulted for assistance with optimizing management of continued agitation on HD 4. Treatment with guanfacine 0.5 mg po tid was initiated with the intention to address underlying anxiety and agitation. After starting guanfacine, the patient only received olanzapine 2.5 mg po (prn for agitation) and olanzapine 2.5 mg iv (prn for agitation) on HD 4. On HD 5, she received olanzapine 2.5 mg po once (prn for anxiety/screaming) and olanzapine 2.5 mg iv three times (prn for anxiety/screaming/agitation). By HD 6, she only required olanzapine 2.5 mg iv twice (prn for anxiety/screaming) prior to her discharge. Despite continued shouting out for her sister at times even when her sister was at her bedside, the patient became generally more pleasant and cooperative, sufficiently enough to allow her to be discharged back to the rehabilitation facility. No significant changes in blood pressure, heart rate, or QT interval were noted after the initiation of guanfacine. Per chart review, guanfacine was continued upon discharge and then the patient was admitted to a hospital again for acute kidney injury after a month, but behavioral disturbances were not noted as a chief concern.

### Case 2

An 85‐year‐old female with a history of hypertension, hyperlipidemia, gastroesophageal reflux disease, atrial fibrillation (on apixaban), coronary artery disease, chronic kidney disease, meningiomas, and dementia presented to the emergency department with worsening confusion, agitation, and combative behavior. Her daughter noted these symptoms had progressively worsened over the past 3 to 4 months, with increasing sundowning, violence, and confusion. A few days before the presentation, the patient had wandered out of the house, believing she was returning to her home, which she no longer owned.

The patient had been diagnosed with mild cognitive impairment 3 years prior, with a mini–mental state examination score of 19/30 and brain MRI showing multiple meningiomas, some volume loss in the parietal lobe, but no hippocampal atrophy. Over the following months, her cognitive decline worsened, and she was diagnosed with mild‐to‐moderate dementia, likely of vascular or Alzheimer's type. This was after she had begun struggling with tasks, such as managing bills and leaving burners on, which triggered fire alarms at home. She had multiple hospital admissions for acute agitation, confusion, and attempts to leave her daughter's home. In the emergency room, the patient became agitated and was administered haloperidol 1 mg iv twice, with minimal effect. Medical workups, including lab tests (CBC, BMP, liver function tests, UA, lipase) and a chest X‐ray, were unremarkable. A head CT showed chronic microvascular changes, a chronic lacunar infarct in the right frontal lobe, and multiple meningiomas, consistent with the previous brain MRI findings. The patient refused to engage in a formal attention assessment, but her presentation appeared more consistent with an advanced‐stage major neurocognitive disorder rather than delirium.

During the encounter, the patient believed she was at her own home and asked the team how staff members had accessed her place. She often became agitated and combative, swinging equipment in the room and wandering the hallways, and was unable to be verbally redirected. Intramuscular olanzapine was administered, and psychiatry was consulted to assist with the management of her behavioral disturbances. At that time, the patient was taking apixaban 5 mg bid, aspirin 81 mg qd, vitamin B_12_ 1000 mcg qd, famotidine 20 mg qd, losartan 25 mg qd, and trazodone 75 mg qhs, all of which she had been taking prior to the admission.

Guanfacine 0.5 mg bid was initiated on HD 1 to help address anxiety and agitation in the setting of her confusion, and trazodone was suspended due to concerns about anticholinergic effects that may worsen the patient's mental status. On HDs 1 and 2, the patient required quetiapine up to 37.5 mg po (prn 25‐mg and 12.5‐mg doses for anxiety) and olanzapine 2.5 mg im (prn dose for agitation) and then quetiapine 25 mg po (prn dose for anxiety) and olanzapine up to 10 mg im (prn 5‐mg and 2.5‐mg doses for agitation), respectively. On HDs 3 and 4, she received olanzapine 2.5 mg im (prn for agitation) and one dose of quetiapine 25 mg po (prn for anxiety) daily. On HD 5, guanfacine was titrated to 1 mg bid, and the patient no longer required any olanzapine, only receiving two prn doses of quetiapine 25 mg po for anxiety daily until her discharge on HD 9. Although the patient still remained confused and irritable at times, claiming she was at home and yelling at staff to leave, she became more redirectable and was amenable to taking oral medications. There was no significant or consistent change in her blood pressure, heart rate, or QT interval. She was discharged on guanfacine 1 mg po bid and quetiapine 25 mg bid. Per chart review, the patient was readmitted to a hospital for decompensated heart failure approximately 4 weeks later. It was documented that the outpatient provider had discontinued guanfacine due to concerns about “oversedation” shortly before the admission, though the causal relationship was unclear.

## DISCUSSION

Antipsychotics are commonly used to manage acute agitation in hospital settings but carry significant risks for side‐effects and increased mortality. Therefore, there is a critical need for alternative effective treatments that can reduce the reliance on antipsychotics, as demonstrated in these clinical cases. Alpha‐2 agonists, like guanfacine, modulate alpha‐2A, 2B, and 2C receptors, inhibiting presynaptic norepinephrine release. This results in therapeutic effects related to sedation, analgesia, neuroprotection, cognition, and sympatholysis.^15^ They may be more effective in managing certain BPSD symptoms, such as anxiety, impulsivity, hyperactivity, aggression, and agitation, but less so for symptoms like depression, apathy, delusions, or hallucinations. It has also been proposed that elevated norepinephrine (NE) may be a factor in neurodegenerative disorders associated with dementia.^16^ Elevated NE has increasingly been associated with the pathophysiology of both AD and Parkinson's disease.^16^ NE may also play a key role in sundown syndrome, with a diurnal buildup of NE correlating with the emergence of neuropsychiatric symptoms in the evening.^16^

Fitzgerald suggested the potential role of five key neuromodulators in AD: norepinephrine, serotonin, dopamine, acetylcholine, and melatonin.^17^ It is proposed that each of these molecules exhibits a U‐shaped dose–response curve, where both insufficient and excessive signaling may contribute to pathology in AD. NE functions in opposition to the other four neuromodulators, and the optimal physiological state is characterized by a balanced interaction between noradrenergic tone and the other modulators.^17^ Most cases of AD, and possibly other diseases, involve an excess of noradrenergic tone and a collective deficit of the other four modulators.^17^ If this hypothesis is true, alpha‐2 agonists, as the drug that reduce noradrenergic transmission, may have a potential role in more effectively preventing or treating AD.^17^

The major limitation of the use of alpha‐2 agonists includes cardiovascular‐related side‐effects, including hypotension and bradycardia. However, guanfacine is much more selective for alpha‐2 receptors, leading to fewer cardiovascular‐related effects.^15^ In a retrospective study, guanfacine was found to be well‐tolerated with rare cardiovascular adverse events when used for managing delirium.^18^ The patients in our cases reported here did not experience significant change in their heart rate or blood pressure after initiating guanfacine. Elderly patients are especially at increased risk of cardiovascular effects due to age‐related physiological changes, so this is a great advantage when considering treatment with guanfacine. Patients with significant QT prolongation (mean 533.17 ms) were treated successfully with guanfacine, without further worsening of QT intervals.^18^ Our cases also showed that guanfacine did not significantly affect the QT interval.

Guanfacine has been shown to deactivate microglia, reduce inflammation, increase neuroplasticity, and enhance cognition through efficient neurotransmission in prefrontal cortex circuits.^19^ Consistent with the prior report, the cases presented here suggested therapeutic effects of guanfacine are observed at low doses (1–2 mg daily), with rapid onset and a reduction in the use of antipsychotic medications, especially in intramuscular or intravenous formulations. In both cases, the use of guanfacine may have prevented prolonging the hospital length of stay, which would have increased the risk of iatrogenic complications, such as infection and delirium.

There are several limitations to these cases. We could not perform the formal attention assessment due to the patients' lack of participation, preventing us from ruling out delirium with confidence. However, the onset of symptoms, the absence of clear waxing and waning pattern in mental status, and the lack of any identifiable acute underlying medical pathology were more consistent with the progression of underlying major neurocognitive disorder. We cannot conclusively state that guanfacine was responsible for the improvement in behavioral disturbances, as antipsychotic medications were also used concurrently. Nevertheless, we observed a reduction in the total amount of antipsychotics required for acute symptom management, which correlated with the initiation or up titration of guanfacine.

## CONCLUSION

Guanfacine may be an effective and well‐tolerated alternative for managing behavioral disturbances in patients with dementia, potentially reducing the need for antipsychotics and the risk of associated side‐effects and offering safer management for the patients. Further research is needed to assess the efficacy and safety of guanfacine for this patient population.

## AUTHOR CONTRIBUTIONS

All authors participated in discussing, writing this manuscript, and revisions. All read and approved the final version of the manuscript.

## CONFLICT OF INTEREST STATEMENT

The authors declare no conflicts of interest.

## ETHICS APPROVAL STATEMENT

This study complied with the CARE guidelines for case reports. For the off‐label use of guanfacine, we explained the expected effects and possible adverse events in detail to the patients and their families and obtained their consent before prescribing the drug.

## PATIENT CONSENT STATEMENT

No written or verbal consent was obtained due to the patients’ mental status limiting their ability to provide consent.

## CLINICAL TRIAL REGISTRATION

N/A.

## Data Availability

Data sharing is not applicable to this article as no datasets were generated or analyzed during the current study.
